# Environmental Gradients Shape Fungal Diversity and Functional Traits in Arctic Biocrusts

**DOI:** 10.3390/jof11120847

**Published:** 2025-11-28

**Authors:** Mia Rümenapp, Burkhard Becker, Ekaterina Pushkareva

**Affiliations:** Department of Biology, Botanical Institute, University of Cologne, Zuelpicher Str. 47B, 50674 Cologne, Germanyb.becker@uni-koeln.de (B.B.)

**Keywords:** biocrust, fungi, Arctic, metagenomics

## Abstract

Arctic biological soil crusts (biocrusts) are known to host diverse fungal communities that facilitate nutrient cycling and soil stabilisation in these harsh environments. In this study, the diversity and composition of fungi were assessed across elevation and spatial gradients in biocrusts from Kongsfjorden (Svalbard) using metagenomic sequencing. Within the observed fungal phyla, Ascomycota was dominant across all sites, with Basidiomycota and Rozellomycota also exhibiting high abundances. Furthermore, saprotrophic fungi were most abundant, followed by mycorrhizal and parasitic guilds. Lichen-associated fungi were also detected across the samples, although their read counts were substantially lower. Additionally, the fungal genus richness and guild composition exhibited no significant variation between elevations, but location within the fjord strongly shaped community structure.

## 1. Introduction

Arctic biological soil crusts (biocrusts) play crucial roles in harsh environments, with fungi contributing significantly to nutrient cycling and soil stabilization [1]. Fungi in biocrusts decompose organic matter, mineralize nutrients, and form symbiotic relationships with phototrophic organisms, impacting both biocrust productivity and soil structure [2]. Arctic fungi exhibit remarkable adaptations, possessing cold-active enzymes, melanized cell walls for UV resistance, and specialized water retention strategies [3,4]. These traits enhance their survival under environmental stress and shape community composition along altitude and environmental gradients.

While studies have documented the responses of phototrophic biocrust members to the variation in altitude, the patterns and drivers of fungal diversity and function across elevation gradients in the Arctic remain poorly understood. We hypothesize that environmental variation along both elevational and spatial gradients, reflecting differences in temperature, moisture, and nutrient availability, will shape the composition and functional potential of Arctic biocrust fungi. In particular, we expect that biocrusts exposed to more extreme conditions will favor fungal taxa and metabolic pathways associated with enhanced stress tolerance and greater metabolic flexibility. By integrating community composition with functional trait analysis, our aim is to provide new insights into how Arctic fungi contribute to biocrust resilience and ecosystem functioning under varying environmental conditions.

## 2. Materials and Methods

Site descriptions, sampling procedures, chemical parameters, and phototrophic community composition were previously described in [5], and a summary is presented in Supplementary Table S1. In summary, biocrust samples were collected during the summers of 2022 and 2023 from three localities in Kongsfjorden (Svalbard), selected to represent a spatial gradient of increasing distance from the open sea, corresponding to a decline in marine influence along the fjord, from the outer, more maritime sites toward the inner, more terrestrial parts of Kongsfjorden: Outer Fjord (OF; Geop, Knau, Kn), Mid Fjord (MF; Knud, Bl), and Inner Fjord (IF; Gr, Os), each comprising low (34–52 m a.s.l) and high (142–354 m a.s.l.) elevation sites (marked as L and H, respectively). The most common vascular plants included *Dryas octopetala*, *Salix polaris*, *Saxifraga oppositifolia*, *Carex* sp., *Silene acaulis*, and *Bistorta vivipara*. The collected biocrusts were well-developed and composed by lichens and bryophytes. Five replicates were collected from each site at both elevations and the total DNA was extracted and subsequently sequenced using the Illumina NovaSeq6000 platform (PE150). The raw reads were submitted to the Sequence Read Archive (SRA) under the project PRJNA1124630.

Bioinformatic analysis was performed using the OmicsBox software (version 3.5.2; Biobam, Valencia, Spain), and the reads were quality-filtered using Trimmomatic [5]. The taxonomic classification of the fungi was executed using the UNITE database (version 10.0). Fungi were further divided into the functional guilds based on their primary lifestyle using the FungalTraits database [6].

For the further functional analysis, all metagenomic reads were classified using Kraken2 (version 2.1.3 [7]) with the RefSeq (2024-11 WGS) database. Sequences assigned to the kingdom Fungi were further extracted. Genes were predicted with MetaEuk (version 7) on the Galaxy platform (e-value = 1 × 10^−5^) and functionally annotated using EggNOG (version 5.0.2 [8]). Differential abundance of KEGG metabolic pathways between low- and high-elevation samples was assessed with a false discovery rate (FDR) cut-off of 0.05 and a log fold change threshold of −2 to +2, where fold change corresponds to the log_2_ ratio of normalized pathway abundance between the two elevation groups.

Statistical analyses were performed in R (version 4.2.1). To test for differences in fungal abundance (number of reads) and genus richness (number of genera) among sampling sites, one-way and two-way analyses of variance (ANOVA) were conducted, followed by Tukey’s HSD post hoc test (*p* < 0.05). Normality of variance was assessed using Shapiro–Wilk’s test. If necessary, data were Log or SQRT transformed. Furthermore, to visualize differences in fungal community composition, non-metric multidimensional scaling (NMDS) was performed using the package *vegan* (version 2.6-4 [9]) and statistical difference was tested with the ANOSIM test. Soil parameters were fitted into the ordination space using the function *envfit*, and their associations with fungal community composition were assessed based on 999 random permutations.

## 3. Results and Discussion

Metagenomic reads (597k) were classified into 19 fungal phyla according to the UNITE database. The majority of these reads in all samples were assigned to the Ascomycota across the samples (40–81%; Supplementary Figure S1). Basidiomycota (3–29%) and Rozellomycota (3–18%) were also the dominant fungal phyla, as found in previous studies of Arctic biocrusts [10]. The relative abundance of the majority of fungal phyla demonstrated no significant variation across elevation and location in the fjord. However, two phyla, namely Mucoromycota and Zoopagomycota, exhibited a marked increase in abundance at lower elevations. It has been demonstrated that certain Mucoromycota fungi facilitate plant growth and benefit from the plant-derived organic matter [11], which is supported by the higher angiosperm coverage observed at the lower sites. The presence of dense vegetation has also been linked to a more diverse soil food web, resulting in the increased populations of microarthropods, nematodes, and protozoa [12]. These organisms, in turn, might serve as prey for numerous species of Zoopagomycota [13].

**Figure 1:**
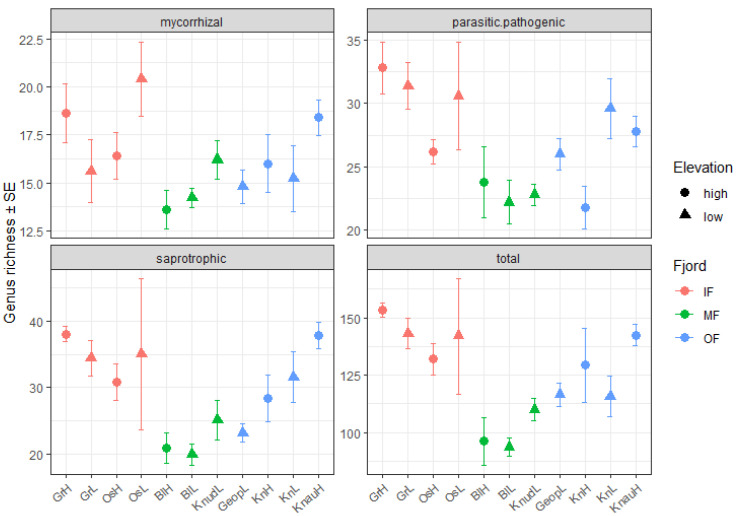
Number of fungal genera per ecological guild based on their primary lifestyle. “Total” represents the total number of fungal genera. Different letters indicate significant differences among locations within the fjord (*p* < 0.05). No significant differences were detected between low and high elevations.

**Figure 2:**
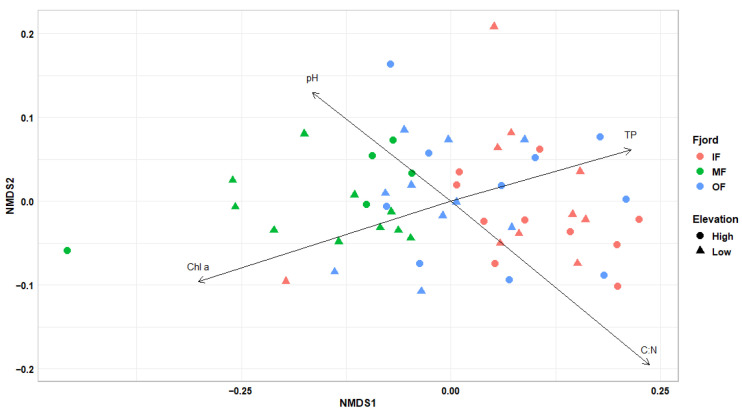
Non-metric multidimensional scaling (NMDS) plot based on Bray–Curtis dissimilarities of fungal community composition derived from metagenomic sequencing reads. Arrows indicate significant correlations (*p* < 0.05) with environmental variables (Chl a = Chlorophyll a content, TP = Total Phosphorus content and C:N = C:N ratio). NMDS stress = 0.08.

A recent study showed that the diversity of saprotrophs in forest soils from Europe and Iceland declines with elevation [14]. In contrast, in the present study, neither fungal genus richness nor read abundance differed significantly between low- and high-elevation sites for any of the fungal guilds (Figure 1). However, the location within the fjord significantly affected the taxonomic composition of the fungal communities, with the number of fungal genera and read abundance being lower in MF sites and higher in IF sites (Figure 1 and Figure 2).

The NMDS plot based on fungal read counts, followed by a PERMANOVA test, showed that the location within the fjord significantly explained variation in community composition (R^2^ = 13.9%, *p* = 0.001), whereas elevation had a non-significant effect (R^2^ = 2.1%, *p* > 0.05; Figure 2). Fungal community dissimilarities were explained by pH, C:N ratio and chlorophyll a, total phosphorus (TP) contents. Sites in MF had higher pH (6.9–7.4) and chlorophyll a (476–811 mg m^−2^), but lower TP (0.1–0.2 mg kg^−1^) and C:N ratio (13–15), which could relate to lower fungal abundance and richness observed there. Higher chlorophyll a concentration, indicative of greater phototrophic biomass, might reflect potential phototroph–fungus associations, including the formation of lichens or other mutualistic interactions involving nutrient exchange or habitat modification within biocrusts [15]. C:N ratio appears to be a critical factor influencing fungal distribution and competitive success, potentially shaping community composition even across small-scale elevational gradients [16]. Sites in OF and IF, with higher C:N ratios (16–19 and 16–21, respectively) and TP content (0.3–0.5 and 0.4–0.5 mg kg^−1^, respectively), might favor fungal taxa capable of efficiently degrading complex carbon compounds, given that the elevated P availability supports the metabolic and enzymatic processes required for their growth and function [17].

Despite no significant differences between fungal phyla and guilds, 27 genera were identified as indicators of sites at high elevation, while 4 genera were designated as indicators of sites at low elevation (Supplementary Table S2). For example, lichenized fungi (e.g., *Staurothele*, *Acarospora*, *Placopsis*, *Calogaya*, *Pannoparmelia*) were significantly associated with higher-elevation sites, which was also consistent with vegetation analyses showing increased lichen coverage in these areas [18]. Harsh environmental conditions at higher elevations limit vascular plant growth, reducing competition for space and nutrients and allowing lichens to proliferate [19]. Increased lichen diversity at higher elevation could provide more hosts for lichenicolous fungi (*Sclerococcum*, *Endococcus* and *Cercidospora*), which were also indicator taxa of high elevations biocrusts. Although lichenized fungi occur within lichen thalli rather than as free-living soil organisms, lichens represent a major structural and functional component of mature Arctic biocrusts [20], and their mycobionts contribute substantially to the overall fungal diversity and functional potential of these systems. Due to their symbiotic lifestyle and specialization within the lichen thallus, these fungi may play ecological roles distinct from free-living soil fungi in the biocrust. In addition, the detection of thermophilic fungi such as Thermomyces as indicator taxa in the studied polar biocrusts is consistent with previous work showing that solar radiation can create warm microhabitats within exposed substrates, generating short periods with temperatures suitable for thermophilic activity even in cold environments [21].

Functional annotation indicated that the core metabolic pathway, such as oxidative phosphorylation (map00190), was consistently observed in fungal communities across the sites (15–38% of annotated fungal contigs). However, fungi from high-elevation sites exhibited significant enrichment in metabolic pathways related to DNA repair, stress signaling, cell-cycle regulation, protein glycosylation, transcriptional control, and specialized metabolism, reflecting adaptations to additional environmental stressors [22,23] associated with higher elevations, such as increased UV exposure, lower temperatures, and more extreme microclimatic variability compared to lower-elevation sites (Table 1). As some pathways were exclusively detected in high-elevation communities and absent in low-elevation ones, these appeared as extreme fold-change values, which should be interpreted as presence/absence signals rather than precise quantitative differences. When comparing the three fjord localities, significant differences were observed only between the MF and OF, with 34 pathways underrepresented in OF (Supplementary Table S3). No significant differences were detected between the IF and the other localities.

Overall, these findings emphasize that Arctic fungal diversity and function are governed not solely by elevation but by a combination of local biotic and abiotic factors, highlighting their integral role in sustaining biocrust stability, nutrient cycling, and ecosystem resilience in polar environments.

## Figures and Tables

**Table 1:** KEGG pathways significantly over-represented in fungal communities from high-elevation compared to low-elevation biocrusts (logFC = log_2_ fold change; FDR = false discovery rate–adjusted *p*-value).

Category	Pathway	KEGG ID	logFC	FDR
DNA repair & genome stability	Base excision repair	map03410	19.6	0.027
	DNA replication	map03030	19.1	0.027
	Homologous recombination	map03440	19.6	0.027
	Mismatch repair	map03430	19.9	0.027
	Nucleotide excision repair	map03420	19.6	0.027
Stress signaling & regulatory networks	HIF-1 signaling pathway	map04066	19.3	0.027
	Hippo signaling pathway—multiple species	map04392	20.3	0.027
	MAPK signaling pathway	map04010	21.1	0.013
	mTOR signaling pathway	map04150	19.9	0.027
	Phosphatidylinositol signaling system	map04070	20.3	0.027
	Phospholipase D signaling pathway	map04072	19.4	0.027
	Ras signaling pathway	map04014	19.7	0.027
	Wnt signaling pathway	map04310	20.7	0.026
Metabolism & nutrient flexibility	Butanoate metabolism	map00650	19.9	0.027
	Degradation of aromatic compounds	map01220	19.2	0.027
	Folate biosynthesis	map00790	19.2	0.027
	Pantothenate and CoA biosynthesis	map00770	19.2	0.027
	Propanoate metabolism	map00640	19.9	0.027
	Sulfur metabolism	map00920	19.2	0.027
	Synthesis and degradation of ketone bodies	map00072	19.2	0.027
	Monobactam biosynthesis	map00261	19.6	0.027
Cell structure, communication & timing	Cell cycle—yeast	map04111	2.0	0.050
	Circadian entrainment	map04713	19.3	0.027
	Mannose type O-glycan biosynthesis	map00515	19.2	0.027
	N-Glycan biosynthesis	map00510	19.2	0.027
Core gene expression machinery	Basal transcription factors	map03022	19.6	0.027

## Data Availability

The original contributions presented in this study are included in the article/Supplementary Material. Further inquiries can be directed to the corresponding author.

## Supplementary Materials

The following supporting information can be downloaded at https://www.mdpi.com/article/10.3390/jof11120847/s1, Supplementary Table S1. Brief description of the studied sites (a detailed description is provided in [5]). TP and C:N ratio correspond to total phosphorus and carbon-to-nitrogen ratio, respectively. Numbers in parentheses indicate the standard error (SE). “L” and “H” in sample names denote low and high elevations, respectively. Locality refers to the position within the fjord: OF = Outer Fjord, MF = Mid Fjord, IF = Inner Fjord. Supplementary Table S2. Fungal indicator genera across different elevations and locations within the fjord. Supplementary Table S3. Differentially abundant KEGG pathways in fungal communities among fjord localities (logFC = log_2_ fold change; FDR = false discovery rate–adjusted *p*-value). No significantly enriched pathways were detected between IF and MF or IF and OF. Supplementary Figure S1. Relative abundance of fungal (a) phyla and (b) functional guilds in biocrusts based on ITS reads generated by metagenomic sequencing. Supplementary Figure S2. Venn diagrams showing the number of common fungal genera across different (a) elevations and (b) locations within the fjord.
