# Supplementary material for: Environmental Gradients Shape Fungal Diversity and Functional Traits in Arctic Biocrusts
Source: J Fungi (Basel). 2025 Nov 28;11(12):847. doi: 10.3390/jof11120847 (PMC12734112; doi:10.3390/jof11120847)
Supplement: Supplementary file 1 [file jof-11-00847-s001.zip › jof-3908590-supplementary.pdf]

Supplementary Table S1. Brief description of the studied sites (a detailed description is provided in [5]). TP and C:N ratio correspond to total phosphorus and carbon-to-nitrogen ratio, respectively. Numbers in parentheses indicate the standard error (SE). “L” and “H” in sample names denote low and high elevations, respectively. Locality refers to the position within the fjord: OF = Outer Fjord, MF = Mid Fjord, IF = Inner Fjord.

Sample	Elevation, m a.s.l.	Locality	pH	Chlorophyll *a*, mg m^{-2}	CN ratio	TP, mg kg^{-1}	Number of ITS fungal reads	
							Total	Mean
GeopL	49	OF	6.1 (0.1)^{bc}	454 (14)^{b}	15.1 (0.3)^{cd}	0.18 (0.02)^{de}	39966	7993
KnudL	48	MF	6.9 (0.3)^{ab}	476 (93)^{b}	15.3 (0.8)^{cd}	0.19 (0.02)^{de}	36423	7285
KnauH	263	OF	7.0 (0.1)^{a}	493 (40)^{b}	18.9 (0.5)^{abc}	0.30 (0.01)^{cd}	63932	12786
GrL	34	IF	6.9 (0.2)^{ab}	110 (19)^{c}	18.7 (0.5)^{abc}	0.51 (0.02)^{ab}	74805	14961
GrH	142	IF	5.3 (0.1)^{d}	59 (9)^{c}	21.3 (0.8)^{a}	0.41 (0.01)^{bc}	81637	16327
OsL	46	IF	6.6 (0.1)^{ab}	164 (33)^{c}	15.5 (0.6)^{cd}	0.38 (0.05)^{c}	80266	16053
OsH	354	IF	5.7 (0.3)^{cd}	129 (31)^{c}	16.9 (2.0)^{abcd}	0.38 (0.02)^{c}	53973	10795
BlL	52	MF	7.4 (<0.1)^{a}	811 (60)^{a}	15.3 (1.1)^{cd}	0.14 (0.01)^{e}	31920	6384
BlH	352	MF	7.1 (0.1)^{a}	636 (30)^{ab}	13.0 (0.3)^{d}	0.15 (0.02)^{e}	32426	6485
KnL	42	OF	7.1 (0.2)^{a}	65 (10)^{c}	19.9 (0.6)^{ab}	0.54 (0.03)^{a}	58486	11697
KnH	219	OF	7.0 (0.1)^{a}	86 (9)^{c}	15.7 (1.2)^{bcd}	0.53 (0.04)^{a}	44427	8885

Supplementary Table S2. Fungal indicator genera across different elevations and locations within the fjord.

Elevation		Fjord		
**High**	**Low**	**IF**	**MF**	**OF**
Wilcoxina	Lecanora	Acidea	Asteromassaria	Geopora
Thermomyces	Meyerozyma	Bacillicladium		Hydropunctaria
Suillus	Piptocephalis	Calogaya		Nowakowskiella
Staurothele	Xerocomus	Candida		Physocladia
Serendipita		Cercidospora		Rhizoclosmatium
Scoliciosporum		Chrysodisca		Viridothelium
Sclerococcum		Cladophialophora		
Pulchromyces		Craterellus		
Placopsis		Dactylospora		
Phyllactinia		Diaporthe		
Phaeomoniella		Epibryon		
Phaeohelotium		Geranomyces		
Pezicula		Hygrocybe		
Pannoparmelia		Ionomidotis		
Ionomidotis		Orbilia		
Hypoxylon		Pannoparmelia		
Hyalorbilia		Pectenia		
Humicola		Pezicula		
Epibryon		Phaeohelotium		
Endococcus		Phaeomoniella		
Dactylospora		Phyllozyma		
Cladosporium		Pithya		
Cladophialophora		Pulchromyces		
Cercidospora		Rhizophydium		
Calogaya		Rhizopus		
Botryosphaeria		Sarcoleotia		
Acarospora		Sarea		
		Sclerococcum		
		Sclerotinia		
		Scoliciosporum		
		Spiromyces		
		Staurothele		
		Suillus		
		Thermomyces		
		Vexillomyces		
		Wilcoxina		

Supplementary Table S3. Differentially abundant KEGG pathways in fungal communities among fjord localities (logFC = log_2 fold change; FDR = false discovery rate–adjusted p-value). No significantly enriched pathways were detected between IF and MF or IF and OF.

Category	Pathway	KEGG ID	logFC	FDR
Under-represented in OF compared to MF				
DNA repair & genome stability	Base excision repair	map03410	-18.7	0.022
	DNA replication	map03030	-18.1	0.022
	Homologous recombination	map03440	-18.7	0.022
	Mismatch repair	map03430	-18.9	0.016
	Nucleotide excision repair	map03420	-18.7	0.022
Stress signaling & regulatory networks	MAPK signaling pathway – yeast	map04011	-1.2	0.022
	Ras signaling pathway	map04014	-18.7	0.014
	Phosphatidylinositol signaling system	map04070	-18.3	0.014
	Phospholipase D signaling pathway	map04072	-18.5	0.014
	Sphingolipid signaling pathway	map04071	-1.4	0.023
	Inositol phosphate metabolism	map00562	-19.1	0.014
Metabolism & nutrient flexibility	Folate biosynthesis	map00790	-18.2	0.022
	Pantothenate and CoA biosynthesis	map00770	-18.2	0.022
	Butanoate metabolism	map00650	-18.9	0.016
	Propanoate metabolism	map00640	-18.9	0.016
	Degradation of aromatic compounds	map01220	-18.2	0.022
	Alanine, aspartate and glutamate metabolism	map00250	-19.6	0.002
	Glycine, serine and threonine metabolism	map00260	-19.5	0.002
	Fatty acid metabolism	map01212	-20.1	0.002
	Arginine biosynthesis	map00220	-19.1	0.014
	Sulfur metabolism	map00920	-18.2	0.022
	Biosynthesis of unsaturated fatty acids	map01040	-18.6	0.020
	Porphyrin and chlorophyll metabolism	map00860	-18.2	0.022
	Starch and sucrose metabolism	map00500	-18.9	0.014
	Fructose and mannose metabolism	map00051	-19.3	0.002

	Galactose metabolism	map00052	-18.6	0.020
	Pentose and glucuronate interconversions	map00040	-18.3	0.014
	Amino sugar and nucleotide sugar metabolism	map00520	-18.9	0.019
Cell structure, communication & timing	Mannose type O-glycan biosynthesis	map00515	-18.2	0.022
	N-Glycan biosynthesis	map00510	-18.2	0.022
	Lysosome	map04142	-19.1	0.014
Core gene expression machinery	Basal transcription factors	map03022	-18.7	0.022
	Ribosome biogenesis in eukaryotes	map03008	-18.6	0.014
	Proteasome	map03050	-18.9	0.020

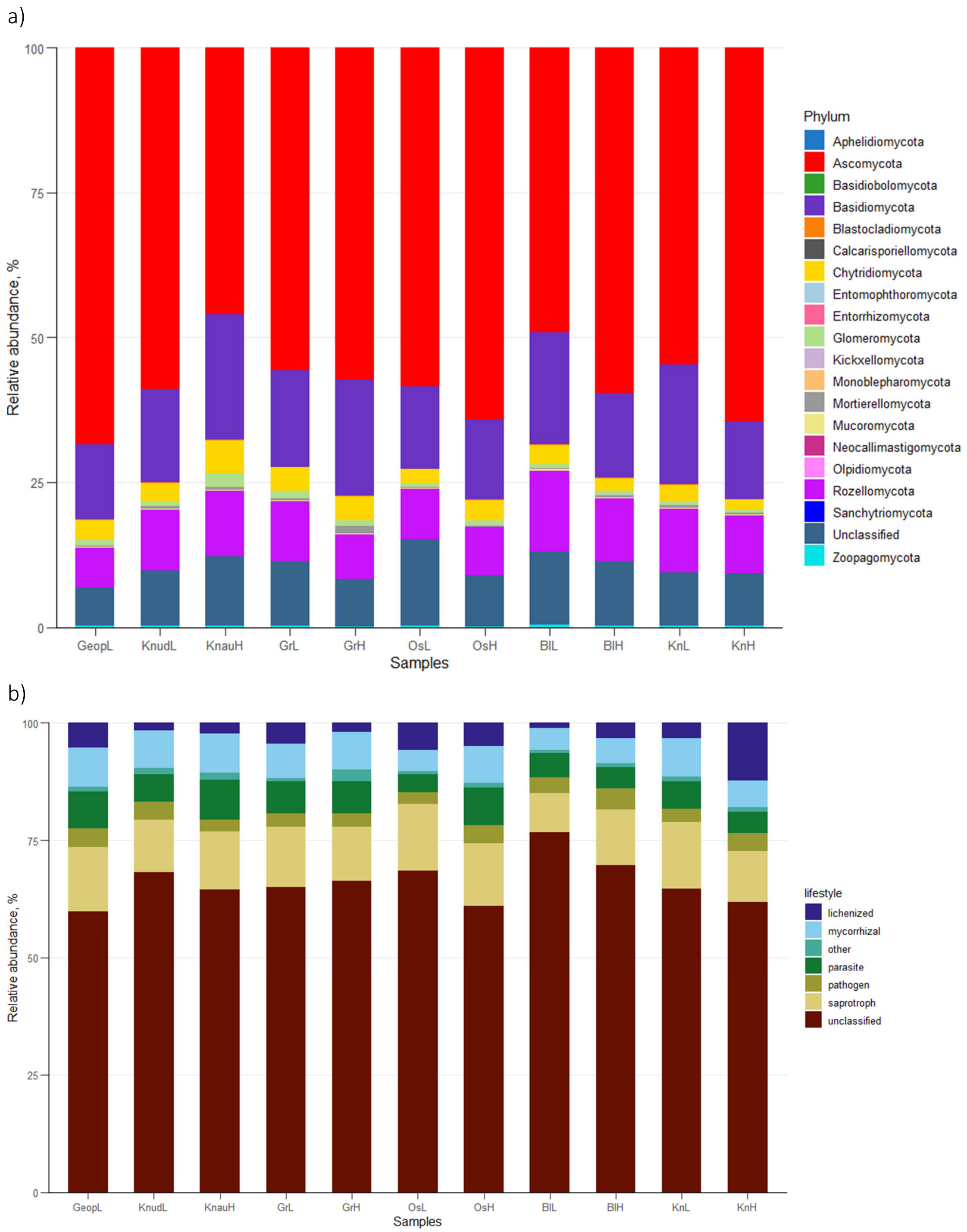

Supplementary Figure S1. Relative abundance of fungal (a) phyla and (b) functional guilds in biocrusts based on ITS reads generated by metagenomic sequencing.

a)

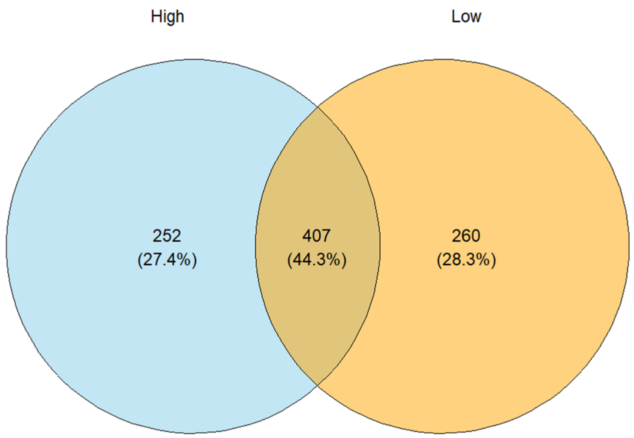

b)

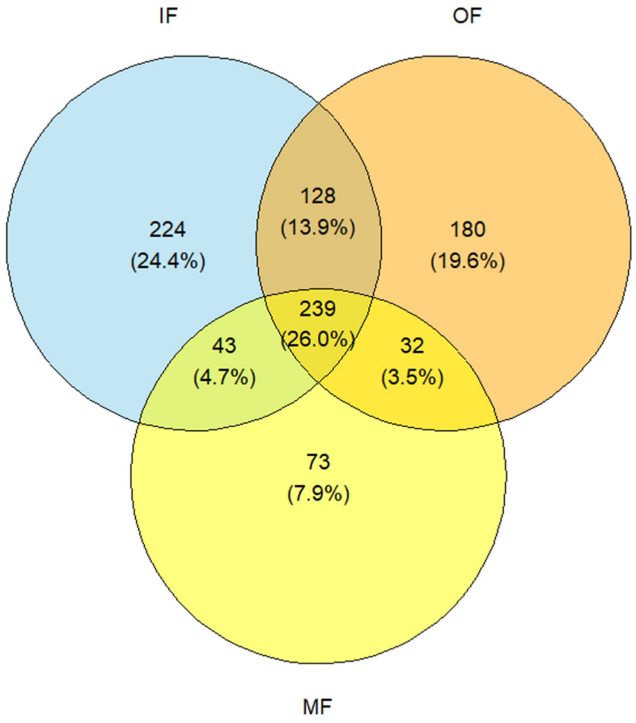

Supplementary Figure S2. Venn diagrams showing the number of common fungal genera across different (a) elevations and (b) locations within the fjord.
